# Dr. Drake on the Diseases of the South

**Published:** 1844-08

**Authors:** 


PROFESSOR DRAKE ON THE DISEASES OF THE SOUTH.

We beg leave to invite the attention of our readers to the letter of Professor Drake, published in another part of the Journal, asking communications from the practitioners of the South concerning the diseases of their region of country. In a letter to us a few days since, written on his way to St. Louis, Professor D. says: "I am now leaving the South, having in two visits devoted myself with some diligence, for six months, to the study of its diseases; and I feel that I can hereafter speak of them, in my lectures, with a degree of confidence which I could not do before. Y.

